# Simplified Assessment of Antiretroviral Adherence and Prediction of Virological Efficacy in HIV-Infected Patients in Cambodia

**DOI:** 10.1155/2010/142076

**Published:** 2009-12-31

**Authors:** Olivier Segeral, Yoann Madec, Boroath Ban, Vara Ouk, Chan Roeurn Hak, Clotilde Le Tiec, Eric Nerrienet, Cécile Goujard, Anne Marie Taburet, Jean Francois Delfraissy, Arnaud Fontanet

**Affiliations:** ^1^Service de Médecine Interne, Centre Hospitalier Universitaire de Bicêtre, AP-HP, 94270 Le Kremlin Bicêtre, France; ^2^Unité d'Epidémiologie des maladies émergentes, Institut Pasteur, 75015 Paris, France; ^3^Service de Maladies Infectieuses, Hôpital Calmette, 12100 Phnom-Penh, Cambodia; ^4^Service de Pharmacologie, Centre Hospitalier Universitaire de Bicêtre, AP-HP, 94270 Le Kremlin Bicêtre, France; ^5^Laboratoire VIH/Hépatites, Institut Pasteur, 12100 Phnom-Penh, Cambodia

## Abstract

*Background*. Adherence to antiviral therapy is important for HIV-infected people living in low- and middle-income countries, because of poor access to alternative regimens. *Methods*. We conducted a cross-sectional survey of adherence in Cambodian patients enrolled in the ESTHER program and treated with WHO first-line regimen for at least 6 months. The survey was based on a self-report questionnaire, drug assay, MCV measurement, visual analog scale, and viral load HIV RNA. *Results*. Two hundred fifty-nine patients treated for a median of 16 months participated in the survey. At inclusion in the program, 158 patients (61%) were ARV-naïve. The virological success rate was 71% overall and 81% in previously ARV-naive patients. Considered individually, the measures suggested perfect adherence in 71% to 93% of patients. In multivariate analysis adjusted for sex and therapeutic status before HAART initiation, only the biological markers were associated with virological efficacy. Self-funded treatment before entry to the program was highly predictive of virological failure. *Conclusion*. Adherence was excellent in these Cambodian patients. Biological markers were predictive of virological efficacy. MCV might thus serve as a simple alternative for assessing adherence and predicting virological efficacy among patients receiving AZT- or d4T-based regimens.

## 1. Introduction

Since highly active antiretroviral therapy (HAART) became widely available in industrialized countries, mortality and morbidity among patients living with HIV/AIDS have been substantially reduced [1]. Recently, access to ARV has improved in low- and middle-income countries.In late 2007, three million people in such countries were receiving HAART [2]. Numerous initiatives have shown that countries such as Brazil, Thailand, and Senegal can provide HAART on a large scale by using produced generic drugs [3–5], and that smaller programs can provide HAART in local healthcare centres [6–10]. These cohort studies also demonstrated the efficacy of World Health Organization-(WHO-)recommended first-line HAART regimens, mainly thanks to excellent adherence to treatment [10–12]. 

It is particularly important to assess adherence during HAART programs, mainly owing to the limited availability of alternative regimens [13]. Indeed, poor adherence can lead to the emergence of drug resistance [14–16], notably to first-line nonnucleoside reverse transcriptase inhibitor (NNRTI-)based regimens recommended by WHO. In countries with poor access to laboratory monitoring (CD4 cell count and viral load), it has been suggested that treatment monitoring could be based simply on physical examination and adherence evaluation [17]. Educational and support programs can improve adherence [18–20].

Adherence is difficult to evaluate, however. Self-report questionnaires have been widely used, for their low cost and simplicity, in both industrialized and developing countries [21, 22]. Visual analog scales have also recently been used to assess adherence [23, 24]. However, the accuracy of these methods can be undermined by issues of recall bias and social desirability [24]. Antiretroviral (ARV) drug assay [25–27] and electronic monitoring [28, 29] are more objective but may be too complex and costly for use in developing countries. Macrocytosis, defined as a mean corpuscular volume (MCV) exceeding 100 fL, is common during treatment with AZT- and d4T-containing regimens and has been proposed as an alternative and less expensive way of assessing adherence [30]. 

We therefore conducted a cross-sectional survey of HIV-infected patients receiving NNRTI-based regimens through the ESTHER program in Phnom Penh (Cambodia) in order to identify simpler tools for the identification of nonadherent patients and for the prediction of virological failure. 

## 2. Patients and Methods

### 2.1. Patients

In February 2003, the Cambodian Ministry of Health and the French ESTHER initiative implemented a free treatment program for patients living with HIV/AIDS at Calmette hospital, Phnom Penh. HAART was prescribed according to WHO recommendations (WHO stages III and IV, irrespective of the CD4 cell count, or asymptomatic patients with CD4 cell counts ≤200/*μ*L) both to ARV-naïve patients and to patients having previously paid for ARV themselves. In keeping with national guidelines, first-line therapy consisted of two nucleoside reverse transcriptase inhibitors (NRTIs) plus one NNRTI. The HAART combination was (AZT or d4T)/3TC/efavirenz (EFV) initially, but was then switched to (AZT or D4T)/3TC/nevirapine (NVP) after July 2004, owing to EFV supply problems. Patients were seen in the hospital every month after enrolment, for a physical examination and to record clinical and therapeutic information. On HAART initiation, the patients also entered a therapeutic education program run by nurses. The patients were seen monthly for three months, or more if the educational objectives were not reached (median duration 4.6 months, IQR: 3–6). The adherence study was restricted to patients who had been on HAART for at least six months, to increase the odds that they had met their educational objectives. 

### 2.2. Adherence Assessment

Adherence was assessed in an outpatient clinic. A self-report questionnaire focusing on recent drug intake was administrated. The questionnaire consisted of the following three items: (i) “did you miss any HAART doses during the last four days?,” (ii) “Were you late for any of your intakes by more than two hours during the last four days?,” and (iii) “did you miss any HAART doses last week-end?.” Because of the low educational level of many patients, one-third of the questionnaires were administered by nurses.

A visual analog scale similar to that developed for pain evaluation was also used. The patients were asked to position a cursor between “never” (score 1) and “always” (score 10) in response to the question: “In general, would you say you take your treatment…?.” Any answer different from 10 was considered to represent nonadherence.

EFV and NVP plasma concentrations were measured by using high-performance liquid chromatography (HLPC). Patients were asked to come to the clinic in the morning without having taken their daily dose of NVP, and 12 hours after their last dose of EFV. Patients with EFV and NVP concentrations below 1000 ng/mL and 3000 ng/mL, respectively, were considered nonadherent.

As all the patients took AZT or D4T, those with an MCV of ≤100 fL were considered nonadherent.

A blood sample was collected to determine the CD4 cell count (CyFlow, Partec, Germany) and HIV RNA viral load (ANRS second-generation (G2) real-time RT-PCR) [31].

### 2.3. Statistical Methods

All patients who had at least one adherence assessment were included in the analysis. Among the 341 patients meeting the inclusion criteria (including more than 6 months of HAART), 13 patients (3.8%) had died, 14 (4.1%) had been lost to follow-up, 9 (2.6%) had been directed to others centers, 12 were on an LPV/r-containing regimen, 8 were on a triple NRTI combination, and 25 could not be evaluated. A total of 259 patients were finally included in the analysis.

The association between each measure of adherence and virological failure (defined as >400 HIV RNA copies/mL) was tested for significance by using the Chi-square test. A logistic regression model was used to evaluate the contribution of the different measures of adherence to predict virological failure. All statistical analyses were performed using Stata 8 software (Stata Corporation, College Station; Texas, USA); all tests were two-sided and *P* values <.05 were considered significant.

## 3. Results

Among the 259 patients evaluated, 151 (58%) were male. Median age was 35 years (interquartile range (IQR), 31–41). At inclusion in the program, the median CD4 cell count was 93/*μ*L (IQR, 38–173); 158 patients (61%) were ARV-naïve and 101 (39%) had already taken ARV before entry to the program (dual NRTI therapy in 63 cases and a fixed-dose combination of D4T/3TC/NVP in 38 cases). At the time of the evaluation, HAART consisted of AZT/3TC/NVP in 183 (71%) patients, AZT/3TC/EFV in 46 patients (18%), d4T/3TC/NVP in 17 patients (6%), and d4T/3TC/EFV in 14 patients (5%). The median CD4 cell count increments at 6 and 12 months were, respectively, 54/*μ*L (IQR, 18–105) and 92/*μ*L (31–144). At the study visit, the median treatment duration was 16.1 months (IQR, 14.3–17.7). Viral load was below 400 HIV RNA copies/mL in 71.5% (*n* = 185) of the patients overall, and in 81% (*n* = 128) of the 158 ARV-naïve patients.

As shown in Table 1, the different measurements showed that most patients were adherent. In particular, 241 patients (93%) had drug concentrations above the thresholds used to define nonadherence. The visual analog scale gave the lowest level of adherence (71%).

The association between virological failure and the different measures of adherence was first investigated in all the patients (Table 2), and then in the previously ARV-naïve patients only (Table 3). Except for the visual analog scale, the different measures were associated with virological failure in univariate analysis, and the association was stronger in ARV-naïve patients. In an attempt to improve the identification of nonadherent patients, we tested various combinations of the measures of adherence. A patient was considered nonadherent if any single measure indicated they were nonadherent. When added to the self-report questionnaire, MCV and drug assay each strengthened the association with the virological response. 

After adjustment for sex and therapeutic status at entry to the ESTHER program, logistic regression showed that the results of the self-report questionnaire did not correlate with virological failure. Only the biological measures were associated with virological failure (Table 4). Exposure to ARV before entry to the ESTHER program was strongly associated with virological failure.

## 4. Discussion

Resistance to NNRTI-based regimens emerges rapidly when adherence is poor. In our study, adherence was considered “perfect” in more than 90% of patients, whatever the method used to estimate it, and 81% of previously ARV-naïve patients were virological responders. Similar good results have been obtained in Cambodia by an MSF team [32, 33], as well as in other low- to middle-income countries with similar regimens [10, 11]. 

Several factors could explain this good adherence. First, treatment was free of charge for all the patients, thanks to the financial stability of the program. Second, good drug procurement and distribution practices avoided drug supply disruption. Both points have been shown to be a significant cause of drug resistance and ARV failure in Uganda [34]. Third, the patients received therapeutic education aimed at improving adherence [19]. The patients had been following this program for between three and six months before being evaluated for virological outcome and simultaneously, for adherence. 

In low- to middle-income countries, where access to viral load assays is limited, HAART efficacy is monitored by using clinical and immunological criteria that have relatively low positive predictive value for virological failure [35, 36]. In order to improve the prediction of virological efficacy, we evaluated different measures of adherence, both individually and in combination. The questionnaire assessing drug intake during the previous four days (two items) and the previous week-end (one item) showed a high level of adherence, as reported elsewhere [21, 33]. Nevertheless, the association with virological failure was weak and was only significant in previously ARV-naïve patients. Moreover, this association disappeared in multivariate analysis. These results may be explained partly by issues of social desirability and the loss of anonymity when the patient needed help from a nurse to answer the questionnaire [25]. 

We also evaluated MCV and plasma drug assay, as more objective measurements of adherence [25, 26]. We found that 93% of patients had drug concentrations within the target range, suggesting a high level of adherence. The favorable pharmacokinetic properties of NNRTIs, which have long half-lives and good oral absorption, ensure consistent drug concentrations and could thus explain this result. Nevertheless, even if the association between virological success and optimal therapeutic drug concentrations was strong in univariate and multivariate analysis, drug assays are difficult to implement in Cambodia, especially in the province where most of patients lived. Furthermore, although drug assay is readily available in low- to middle-income countries, it is unlikely that it would be cost-effective for monitoring HAART.

The absence of macrocytosis was associated with virological failure, as all the patients were on AZT- or d4T-based regimens. The association was strong in previously ARV-naïve patients and persisted in multivariate analysis. In Cambodia, as well as in many other low- to middle-income countries, AZT and d4T are the most widely used NRTIs, in keeping with WHO recommendations. MCV was determined during standard automated blood analyses, which are widely available in many district hospitals in Cambodia. This parameter might thus be used in combination with clinical and immunological criteria to monitor HAART exposure and efficacy. 

Forty percent of our patients had taken dual NRTI therapy or a fixed dose combination of D4T/3TC/NVP before entry to the ESTHER program, and the efficacy of the study regimens was lower in these patients than in previously ARV-naïve patients. Self-funded treatment in the private sector was significantly associated with subsequent virological failure, even after adjustment for other factors [37]. This was confirmed by our logistic regression analysis. These patients probably had NRTI and/or NNRTI resistance mutations (data not shown), and subsequent HAART was often ineffective despite good adherence.

Resistance to NNRTIs and NRTIs is the most important issue in low- to middle-income countries, where access to protease inhibitors is limited. WHO-approved first-line HAART must be maintained as long as possible, and the identification of patients at risk of resistance must be a priority. Unfortunately, access to viral load assay and genotypic resistance tests is currently limited. As self-report questionnaires may lack accuracy and as drug monitoring is difficult to implement, MCV could be an interesting alternative marker of adherence. Viral load assay could be added if MCV results suggest poor adherence.

## Figures and Tables

**Table 1:** Description of the tools used to assess adherence.

	*N* (%)
HAART dose missed in the last four days*	

Yes	5 (2)
No	252 (98)

Dose delayed by >2 hours in the last four days*	

Yes	22 (8.5)
No	235 (91.5)

HAART dose missed the previous weekend*	

Yes	17 (6.6)
No	240 (93.4)

Self-report questionnaire (combining the above three items)*	

At least one missed HAART dose	34 (13.2)
100% adherent	223 (86.8)

Visual analog scale*	

<9	11 (4.3)
9	62 (24.1)
10	184 (71.6)

Antiretroviral drug concentrations (in ng/mL)	

<1000 for EFV or <3000 for NVP	18 (7)
≥1000 for EFV or ≥3000 for NVP	241 (93)

Macrocytosis (MCV > 100 fL)**	

No	23 (9.1)
Yes	229 (90.9)

*Available for 257 patients, **Available for 252 patients.

**Table 2:** Association between the four measures of adherence and virological failure in the overall population.

	*N*	Virological failure: *N* (%)	OR [95% CI]	*P*
Self-report questionnaire (all three items)*				

100% adherent	223	63 (28.2)	1	
At least one missed HAART dose	34	11 (32.3)	1.21 [0.56–2.64]	.62

Visual analog scale*				

10	184	54 (29.3)	1	
9	62	18 (29.1)	0.98 [0.52–1.85]	.70
<9	11	2 (18.2)	0.54 [0.11–2.55]	

Antiretroviral drug concentrations (ng/mL)				

≥1000 for EFV or ≥3000 for NVP	241	61 (25.3)	1	
<1000 for EFV or <3000 for NVP	18	13 (72.2)	7.67 [2.63 –22.40]	<.0001

Macrocytosis (MCV ≥100 fL)**				

Yes	229	62 (27.1)	1	
No	23	12 (52.1)	2.94 [1.23–7]	.015

Self-report questionnaire plus macrocytosis				

100% adherent	200	56 (28)	1	
At least one mistake	50	18 (36)	1.44 [0.75–2.78]	.27

Self-report questionnaire plus drug assays				

100% adherent	209	54 (25.8)	1	
At least one mistake	48	20 (41.7)	2.05 [1.07–3.93]	.031

OR: odds ratio; CI: confidence interval, *Available for 257 patients, **Available for 252 patients.

**Table 3:** Association between the four measures of adherence and virological failure in the previously HAART-naïve population.

	*N*	Virological failure: *N* (%)	OR [95% CI]	*P*
Self-report questionnaire (all three items)*				

100% adherent	134	22 (16.4)	1	
At least one missed HAART dose	22	8 (36.4)	2.91 [1.09–7.76]	.034

Visual analog scale*				

10	110	20 (18.1)	1	
9	39	8 (20.5)	1.16 [0.46–2.90]	.78
<9	7	2 (28.6)	1.8 [0.32–9.95]	

Antiretroviral drug concentrations (ng/mL)				

≥1000 for EFV or ≥3000 for NVP	149	24 (16.1)	1	
<1000 for EFV or <3000 for NVP	9	6 (66.7)	10.42 [2.43–44.54]	.002

Macrocytosis (MCV ≥ 100 fL)**				

Yes	141	23 (16.3)	1	
No	14	7 (50)	5.13 [1.64–16.02]	.007

Self-report questionnaire and macrocytosis				

100% adherent	121	18 (14.9)	1	
At least one mistake	32	12 (37.5)	3.43 [1.43–8.22]	.006

Self-report questionnaire and drug assays				

100% adherent	129	20 (15.5)	1	
At least one mistake	27	10 (37)	3.20 [1.28–8]	.015

OR: odds ratio; CI: confidence interval, *Available for 156 patients, **Available for 155 patients.

**Table 4:** Multivariate logistic regression: factors associated with virological failure (*N* = 250).

	OR [95% CI]	**P**
Sex		

Female	1	
Male	0.59 [0.32–1.09]	.094

Previously treated patients		

No	1	
Yes	3.98 [2.15–7.36]	<.0001

Self-report questionnaire		

100% adherent	1	
At least one mistake	0.88 [0.36–2.14]	.77

Macrocytosis		

Yes	1	
No	3.09 [1.17–8.18]	.023

Plasma drug concentrations		

≥1000 for EFV or ≥3000 for NVP	1	
<1000 for EFV or <3000 for NVP	10.46 [3.06–35.78]	<.0001

OR: odds ratio; CI: confidence interval.
